# GOING OUTDOORS AND COMPUTER USE PREDICT CHANGES IN SELF-RATED CHEERFULNESS OF OLDER ADULTS OVER 10 YEARS

**DOI:** 10.1093/geroni/igae098.2902

**Published:** 2024-12-31

**Authors:** Yufang Tu, Melissa O’Connor, Rachel Grace

**Affiliations:** North Dakota State University, Fargo, North Dakota, United States; North Dakota State University, Fargo, North Dakota, United States; North Dakota State University, Fargo, North Dakota, United States

## Abstract

Positive affect decreases mortality risks and enhances wellness and longevity among older adults. Research indicates that adopting digital technology increases social inclusion among older individuals while going outdoors may benefit physical and psychological health. The current study explored whether the frequency of going outside and computer ownership/use relate to changes in older adults’ cheerfulness over ten years. Data from the first 11 waves (2011-2021) from the National Health and Aging Trends (NHATS) Study were used. The study included 35,687 participants at baseline. Self-rated cheerfulness on a 5-point scale was measured. Longitudinal multilevel modeling was conducted with the frequency of going outside of home as a time-variant predictor (5-point scale) and computer ownership as a time-invariant predictor (yes/no). Covariates included sex, age groups, education level, and self-rated health. Interactions between time and go-out frequency, and time and computer ownership/use were also examined. The effect of go-out frequency on cheerfulness depends on different time points within (Estimate within=-0.01, p=0.002) and across individuals over time (Estimate between= -0.01, p=0.008). The trajectory of cheerfulness for individuals without computer use did not change over time (p=0.810). However, the cheerfulness of those with computer use did change over time (p=0.023). This study found that the go-out frequency and computer ownership/use predict changes in and improve older adults’ cheerfulness over ten years, beyond demographic and health factors. This suggests the potential to include outdoor activities and computer adoption in efforts to promote healthy aging.

